# La prostatectomie radicale: résultats carcinologiques au Centre Hospitalier Universitaire Aristide Le Dantec

**DOI:** 10.11604/pamj.2021.38.56.25198

**Published:** 2021-01-18

**Authors:** Ndiaga Seck Ndour, Yaya Sow, Babacar Sine, Modou Ndiaye, Alioune Sarr, Amath Thiam, Cyrille Zé Ondo, Ndèye Aissatou Bagayogo, Aboubacar Traoré, Samba Thiapato Faye, Abdoulaye Ndiath, Ousmane Sow, Boubacar Fall, Babacar Diao, Alain Khassim Ndoye

**Affiliations:** 1 Centre Hospitalier Universitaire Aristide Le Dantec, Dakar, Sénégal

**Keywords:** Cancer prostatique, prostatectomie radicale, survie, Prostate cancer, radical prostatectomy, survival

## Abstract

Le but était d´évaluer les résultats carcinologiques de la prostatectomie radicale. Il s´agissait d´une étude rétrospective monocentrique au service d´urologie-andrologie de l´Hôpital Aristide Le Dantec de Dakar du 1^er^ juin 2010 au 31 mai 2016. Nous avons colligé 60 cas de prostatectomie radicale par voie rétropubienne associée à un curage ganglionnaire ilio-obturateur. Après la prostatectomie radicale (PR), le taux d’antigène prostatique spécifique (PSA) était indétectable (<0,1 ng/ml) chez 20 patients (33,3%). Onze malades (18,3%) qui avaient une récidive biochimique, ont une hormonothérapie complémentaire. Une réponse a été obtenue après instauration du traitement avec un taux de PSA total redevenu indétectable après un suivi de 8 mois. La durée moyenne de la survie globale était de 17,5 mois avec une médiane de 9,49. Le taux de survie globale cumulé à 1 an, 3 ans et 4 ans étaient respectivement de 42,4, 13,6 et 6,8%. La durée moyenne de la survie sans récidive était de 17,3 mois avec une médiane de survie sans récidive biochimique qui était de onze (11) mois. La durée moyenne de la survie spécifique était de 8,1 mois avec une médiane de 3 mois. Les marges de résection étaient positives chez sept (7) patients soit un taux de 11,6%. Quatre(4) patients avaient un envahissement ganglionnaire. La prostatectomie radicale indiquée dans quelques cas dans notre pratique, est une méthode thérapeutique efficiente avec de bons résultats carcinologiques.

## Abstract (en)

The purpose of this study was to assess outcomes of radical prostatectomy in patients with prostate cancer. We conducted a retrospective single-center study in the Department of Urology and Andrology at the Aristide Le Dantec Hospital in Dakar from June 1, 2010 to May 31, 2016. We collected data of 60 patients undergoing radical retropubic prostatectomy associated with dissection of the iliac and obturator nodes. After radical prostatectomy, prostate specific antigen (PSA) levels were undetectable (<0.1 ng/mL) in 20 patients (33.3%). Eleven patients (18.3%), who had biochemical relapse, received complementary hormone therapy. Patients achieved a response after initiation of treatment, and total PSA became undetectable again after an 8-month follow-up period. Mean overall survival was 17.5 months, with a median of 9.49. Cumulative overall survival rates at 1 year, 3 years and 4 years were 42.4, 13.6 and 6.8%, respectively. Mean relapse-free survival was 17.3 months, with a median biochemical relapse-free survival of eleven (11) months. The mean duration of specific survival was 8.1 months, with a median of 3 months. Seven patients had positive resection margins (11.6%). Four patients had lymph node involvement. Radical prostatectomy, suggested in some patients with prostate cancer in our practice, has been shown to be an effective therapeutic method leading to good outcomes.

## Introduction

Le cancer de la prostate est le plus fréquent des cancers de l´homme et la deuxième cause de décès par cancer [1] La prostatectomie radicale est l´un des traitements de référence du cancer de la prostate localisé [2]. Ce traitement chirurgical est également recommandé pour des cancers à haut risque, localement avancés ou oligo-métastatiques (moins de 3 sites métastatiques) [3-5]. C´est en 1904 que Young a réalisé la première prostatectomie radicale par voie périnéale. Une meilleure compréhension de l´anatomie avec la possibilité de préservation des bandelettes vasculonerveuses a été développée par Walsh [6] au début des années 1980. De la voie ouverte aux techniques mini-invasives, elle a connu une évolution importante afin d´améliorer les résultats carcinologiques et fonctionnels. Dans notre contexte, il s´agit d´une chirurgie encore réalisée à un taux faible du fait de la difficulté de sélection de patients éligibles qui sont vus pour la plupart à un stade avancé de la maladie cancéreuse. L´objectif de notre étude était d´évaluer les résultats carcinologiques de la prostatectomie radicale.

## Méthodes

Dans cette étude ont été inclus soixante (60) patients admis pour cancer de la prostate et ayant une preuve histologique chez qui un traitement radical a été réalisé. Nous avons effectué une étude rétrospective portant sur 60 cas de prostatectomie radicale par voie rétropubienne associée à un curage ganglionnaire ilio-obturateur réalisés au service d´urologie de l´hôpital Aristide Le Dantec. Une prostatectomie radicale élargie aux tissus voisins associée à un curage ganglionnaire étendu était réalisée pour les cancers localement avancés. Les patients sont répartis selon le stade tumoral sur le Tableau 1. Les patients étaient revus à trois mois puis tous les six mois avec un contrôle du taux de PSA total pendant six (6) ans. Les critères d´appréciation des résultats étaient: la cinétique du PSA total, les résultats de l´examen anatomo-pathologique de la pièce opératoire, la survie globale, la survie sans récidive. La progression était définie par un dosage PSA supérieur à 0,1ng/ml ou par l´apparition de métastases. Le délai de progression était estimé pour chaque malade. La survie a été évaluée selon la méthode de Kaplan-Meier.

**Tableau 1:** répartition des patients selon le stade tumoral

Stade tumoral	Nombre de patients	Pourcentage %
Risque faible	3	5
Risque intermédiaire	14	23,3
Risque élevé	21	35
Localement avancés	22	36,6

## Résultats

**Figure 1:**
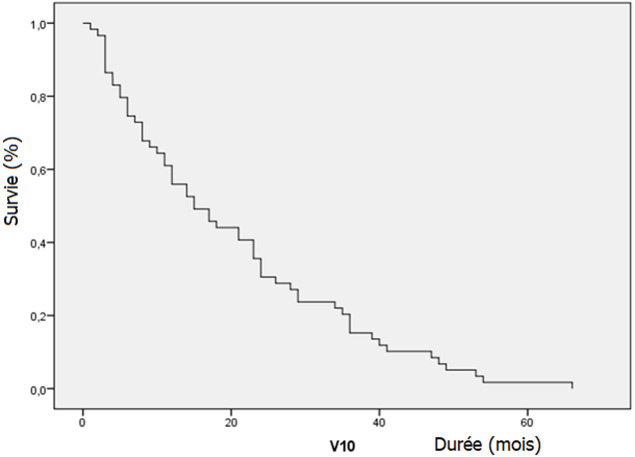
courbe de survie globale après prostatectomie radicale

**Figure 2:**
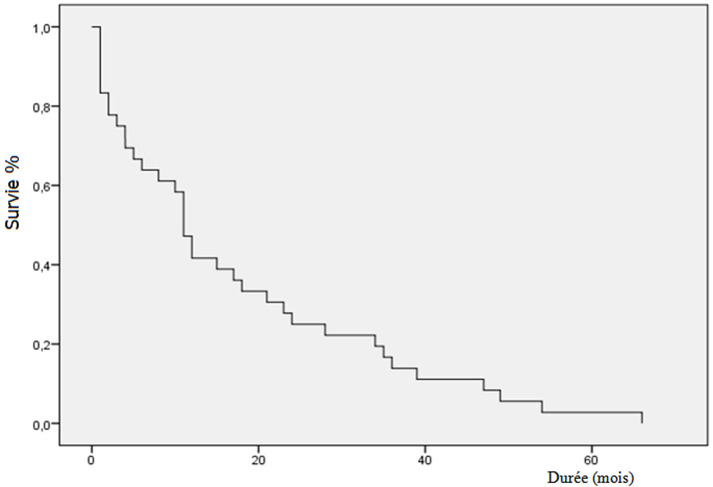
survie sans récidive après prostatectomie radicale

La durée moyenne du suivi était de 20 mois. Le taux de PSA était indétectable (<0,1 ng/ml) chez 20 patients (33,3%). La plupart de ces patients avaient un cancer à haut risque D´Amico ou localement avancé. Onze patients (18,3%) qui avaient une récidive biochimique, ont eu une hormonothérapie complémentaire avec de la goséréline (8 cas) et une pulpectomie bilatérale (3 cas). Une réponse a été obtenue après instauration du traitement avec un taux de PSA total redevenu indétectable. Durant le suivi, 5 patients sont décédés (8,3%), avec deux cas en rapport avec l´évolution du cancer et dans 3 cas décédés d´une autre cause. Les patients décédés appartenaient tous au groupe de cancers à haut risque de D´Amico ou localement avancés. La survie globale des patients à risque faible ou intermédiaire était de 100%. La durée moyenne de la survie globale était de 20,8 mois avec une médiane de 15. Le taux de survie globale cumulé à 1 an, 3 ans et 4 ans étaient respectivement de 79,7, 40,7 et 20,3% (Figure 1). La durée moyenne de la survie sans récidive était de 17,3 mois avec une médiane de survie sans récidive biochimique qui était de onze (11) mois (Figure 2). Les marges de résection étaient positives chez sept (7) patients soit un taux de 11,6% des patients dont la plupart (85%) avaient un cancer localisé à haut risque de D´Amico. Quatre(4) patients avaient un envahissement ganglionnaire.

## Discussion

Après prostatectomie radicale, le PSA devient normalement indétectable en 4 à 6 semaines [7]. Dans notre série, le taux de PSA était indétectable (<0,1 ng/ml) chez 20 patients (33,3%). Une récidive biochimique a été diagnostiquée chez 23 (38,3%) patients dont la majorité avaient un cancer localement avancé ou à haut risque de D´Amico. Delongchamps *et al*. [8] rapportent 52% de récidive biochimique. La majorité des études retrouve 25 à 35% de récidive biologique à dix ans, après prostatectomie totale, tous stades confondus, mais avec des taux inférieurs à 18% en cas de PSA initial inférieur à 10 ng/ml [9]. Le risque de progression du taux de PSA en cas de PSA supérieur à 0,2 ng/ml en postopératoire est de 86% à un an et de 100% à trois ans [10]. En effet Molina *et al*. [11] rapportent que le taux de PSA pré-thérapeutique, le score de Gleason (4+3), le stade pathologique pT3b et des marges chirurgicales positives sont des facteurs pronostiques indépendants liés à la récidive biochimique. Ces résultats seraient en rapport avec le stade avancé des cancers de la prostate dans notre série. Parmi ces patients, onze (47,8%) ont eu une hormonothérapie qui était médicale (analogue LHRH) dans 8 cas et chirurgicale dans 3 cas. Ward *et al*. [12] rapportent 78% de traitement complémentaire (hormonothérapie) pour des cancers localement avancés. La décision du type de traitement de rattrapage doit être prise en fonction des paramètres pathologiques habituels (statut ganglionnaire, marges, score de Gleason, stade pT) ainsi que des paramètres biologiques postopératoires que sont le taux immédiat postopératoire du PSA et sa vélocité. Une forte vélocité du PSA et des caractéristiques pathologiques défavorables orientent plutôt vers une maladie métastatique et une prise en charge systémique de la maladie résiduelle [13].

La durée moyenne de la survie globale était de 20,8 mois avec une médiane de 15 mois. Le taux de survie globale cumulée à 1 an, 3 et 4 ans était estimé respectivement à 71,7%, 40,7 et 20,3%. Cette survie globale était très inférieure à celle rapportée dans les séries occidentales. Li *et al*. [14] rapportent un taux de survie de 41% à 5 ans après prostatectomie radicale. La durée moyenne de survie sans récidive biochimique était de 17,3 mois avec une médiane de 11 mois. Le taux de survie sans récidive (SSR) biochimique cumulé était de 66,7% et 33% respectivement à 12 et 24 mois. Roehl *et al*. [15] rapporte un taux de SSR de 73% et 15% respectivement pour les cancers localisés et les cancers localement avancés après un suivi de 10 ans. Delongchamps [8] rapporte également dans sa série une SSR biochimique de 48% à 5 ans pour des cancers localement avancés. Cette différence serait essentiellement due au fait que nos patients avaient pour la plupart des cancers avancés. En cas de récidive biologique, l´évolution se fait inexorablement vers la progression avec une histoire naturelle évoluant en deux phases: une première phase asymptomatique de huit ans en moyenne jusqu´à l´apparition des métastases, puis une seconde de cinq ans avant le décès [9]. Si le PSA reste indétectable, pendant sept à dix ans en postopératoire, il n´y a plus de risque de récidive et la guérison est affirmée [9].

D´après Roehl *et al*. [15], la valeur du PSA pré-opératoire, le stade tumoral, le score de Gleason et les résultats histologiques de la pièce opératoire seraient des facteurs prédictifs de récidive biologique. En effet Ritch *et al*. [16] rapportent que la population afro-américaine est plus susceptible de développer une récidive biochimique après prostatectomie radicale démontrant le caractère agressif du cancer chez le sujet de race noire. Ces données réconfortent l´idée du dépistage précoce individuel en identifiant les facteurs de risque (âge, origine ethnique africaine) et l´estimation de l´espérance de vie.

Cependant la prostatectomie radicale reste une méthode de choix efficace dans la prise en charge des cancers de la prostate localisés à haut risque D´Amico ou localement avancés. En effet, d´après Vickers *et al*. [17] la prostatectomie radicale apporte un bénéfice avec une diminution du risque relatif de décès par cancer estimé entre 0 et 25% en fonction de l´âge, du score de Gleason et du stade clinique. Les marges de résection étaient positives chez sept (7) patients, soit un taux de 11,6%. Quatre patients (cancers à haut risque) avaient un envahissement ganglionnaire soit un taux de 6,6% tandis que Xylinas *et al*. [18] rapportent un taux de 17 à 31% d´envahissement ganglionnaire. D´après Salomon *et al*. [19], le taux de marges chirurgicales positives (MCP) était de 32,5%, 18,5% et 26,4% respectivement pour les voies rétropubienne, périnéale et laparoscopique. Chez tous nos patients opérés, l´abord était par voie rétropubienne. En effet dans cette voie d´abord, du fait d´une dissection difficile, l´apex est le premier site de MCP [19]. D´après Soloway et Wieder [20] la positivité des marges chirurgicales après prostatectomie radicale est une situation complexe dont la fréquence varie de moins de 10% à plus de 40% selon le stade pathologique et les opérateurs. En revanche, les options thérapeutiques plausibles devant des MCP peuvent varier de la surveillance, la radiothérapie et/ou l´hormonothérapie adjuvante lorsque la récidive biologique et la progression de la maladie sont évidentes [20].

## Conclusion

La prostatectomie radicale est réalisée à un taux encore faible dans notre pratique du fait du retard diagnostique contrairement aux séries occidentales où cette intervention est réalisée de manière courante. Les résultats sont corrélés aux paramètres clinico-pathologiques. Du fait de la prépondérance de formes avancées du cancer, elle est une option thérapeutique convenable pour nos patients. Ces données réconfortent l´idée du dépistage précoce individuel pour une meilleure prise en charge du cancer de la prostate et l´amélioration de la survie spécifique liée au cancer de prostate.

### Etat des connaissances sur le sujet

- Le cancer de la prostate est le plus fréquent des cancers de l´homme et la deuxième cause de décès par cancer;
- La prostatectomie radicale est l´un des traitements de référence du cancer de la prostate localisé qui offre le plus de garantie en terme de contrôle carcinologique;
- La Prostatectomie radicale est le seul traitement ayant montré une amélioration en survie globale et survie spécifique dans le traitement du cancer de prostate localisé.

### Contribution de notre étude à la connaissance

- Ces données réconfortent l´idée du dépistage précoce individuel en identifiant les facteurs de risque (âge, origine ethnique africaine) et l´estimation de l´espérance de vie;
- La prostatectomie radicale reste une méthode de choix efficace dans la prise en charge des cancers de la prostate localisés à haut risque D´Amico ou localement avancés.
